# Co-design of harm reduction materials for people who inject drugs to implement research findings

**DOI:** 10.1186/s12954-019-0300-z

**Published:** 2019-06-07

**Authors:** Deborah Hussey, Zoe Trinder-Widdess, Cassie Dee, Darren Bagnall, Tatty Bojangles, Joanna May Kesten

**Affiliations:** 1 Bristol Drugs Project, 11 Brunswick Square, Bristol, BS2 8PE UK; 2 National Institute for Health Research Collaboration for Leadership in Applied Health Research and Care West (NIHR CLAHRC West), University Hospitals Bristol NHS Foundation Trust, Bristol, UK; 3 Bristol Health Partners, Bristol, UK; 4 Population Health Sciences, Bristol Medical School, University of Bristol, Bristol, UK; 5 National Institute for Health Research Health Protection Research Unit (HPRU) in Evaluation of Interventions, University of Bristol, Bristol, UK

**Keywords:** Low dead space syringes, People who inject drugs, Harm reduction, Peers, Co-design, Involvement, Needle and syringe programmes

## Abstract

**Background:**

Equipment used by people who inject drugs (PWID) either has a needle which is fixed to the syringe or can be detached and replaced. Detachable low dead space syringes (LDSS) have been developed, with less space between the needle and plunger (referred to as dead space) than traditional detachable equipment. This equipment can help protect PWID from harm of infection as less blood is transferred if equipment is shared. Our previous research found that detachable LDSS are likely to be acceptable to PWID, and we produced recommendations for their introduction in needle and syringe programmes (NSP) in the United Kingdom (UK). We held a national stakeholder meeting to discuss how to accelerate the pace and scale of the rollout and uptake of detachable LDSS. This commentary reflects on the involvement of PWID as co-designers of harm reduction materials to implement these research findings in a way that supports the uptake of LDSS equipment by NSP and service users. We present the user-centred design process, peer reflections on the project, and lessons learnt by the team working with the peers.

**Main body:**

Peers and stakeholders translated the research into easy to understand messages following a consultation with NSP across the UK. Working with Linnell Publications over three workshops, peers selected their preferred design style and informed the language, messages, and overall look of the designs. The peers ensured the designs avoided images and language with negative connotations, humour, and unequivocal language. Peers said that they found the process enjoyable and informative—leading to increased awareness of harm reduction practices. The facilitators took steps to ensure the views of the peers were heard throughout. They reflected on the importance of involving PWID meaningfully throughout the project. Without the peers, the designs would be less effective and engaging to their target audience.

**Conclusion:**

We conclude that placing peers at the heart of this research implementation project was essential to ensure the materials are appropriate and engaging and do not stigmatise or alienate the intended audience unintentionally. We recommend that others planning similar work include peers within the entire project to support their meaningful contribution.

## Background

### Why is low dead space equipment important?

Injecting equipment used by people who inject drugs (PWID) either has a needle which is fixed to the syringe or can be detached and replaced. New needles should be used for each injection to ensure they are sharp and sterile. Detachable, longer needles are needed for groin injecting [1]. Changing needles is also important if they become blunt or blocked [1] and when distributing drugs between people [2].

Injecting equipment contains either low or high amounts of ‘dead space’ between the needle and plunger, hereafter referred to as low dead space syringes (LDSS) and high dead space syringes (HDSS). HDSS transfer more blood if re-used [1, 3]. Blood-borne viruses (BBVs), such as Hepatitis C virus (HCV) [4] and human immunodeficiency virus (HIV) [5], survive longer in HDSS than LDSS [4], and the risk of passing these BBVs between people is hypothesised to be higher in HDSS compared to LDSS [5, 6].

In 2016, Exchange Supplies, a social enterprise that supplies injecting equipment, information, and services for PWID, developed the Total Dose dose range of detachable low dead space needles, which significantly reduced the dead space compared to standard detachable needle/syringe combinations [7]. The replacement of equivalent detachable HDSS has the potential to increase the availability of LDSS and reduce the risk of passing viruses if the equipment is shared [5, 6, 8–12].

Our research, conducted in Bristol and Bath, found that detachable LDSS are likely to be acceptable to PWID and we produced recommendations for their introduction in needle and syringe programmes (NSP) in the United Kingdom (UK) [13]. NSP are a key intervention for preventing BBVs among PWID, providing sterile injecting equipment and harm reduction advice. Sixty-one per cent of PWID in England, Northern Ireland, and Wales and 73% in Scotland report adequate needle and syringe provision (defined as the number of needles and syringes meeting or exceeding the number injections) [14]. While in Bristol, 57% of PWID have ‘high’ NSP coverage [15]. In 2011, estimates of the population of PWID in Bristol were 2295 (2025–2564) [15], compared to 93,401 (90,974–96,757) in England [16].

At the time of this project, although detachable LDSS were available and had been rolled out in Wales, the uptake of the product by NSP elsewhere in the UK was at an early stage, with only a relatively small number of early adopters using them. Therefore, there was a need to support the implementation (‘systematic efforts to encourage adoption’ p21 [17]) of this equipment using information materials in line with the recommendations of our research.

### Implementing and co-producing research

The challenge of translating health research evidence into practice is well recognised [18, 19]. Utilising implementation science methods, the ‘how to’ or strategies used to change practice [20] can support the successful uptake of evidence and improvement in health outcomes [21]. Implementation strategies can include policy changes, educating key stakeholders or champions who can bring about change, and engagement with service users and patients [21]. These strategies must support ‘context sensitive’ intervention scale-up [22, 23]. One approach to implementation is co-production which has been defined as ‘an approach in which researchers, practitioners, and the public work together, sharing power and responsibility from the start to the end of the project, including the generation of knowledge’(p4) [24]. The central tenets of co-production are the ‘Sharing of power’, ‘Including all perspectives and skills’, ‘Respecting and valuing the knowledge of all those working together on the research’, ‘Reciprocity’, and ‘Building and maintaining relationships’ [24]. Meaningful co-production requires an understanding of different stakeholder agendas, roles, and motivation [19]. Such understanding takes time and effort to develop and sustain [19]. Co-production and implementation are often adaptive, iterative, and reflective in nature [22, 23]. Underlying the co-production approach is an assumption that those most affected by research, in this case PWID and those who work with them, have the right skills and knowledge to design and deliver it [24].

In this commentary, we present a co-production project designed to implement our research findings [13] to support the uptake and use of LDSS by co-designing harm reduction materials with PWID. We describe a national stakeholder meeting, the user-centred design process, peer reflections on the project and lessons learnt by the team working with the peers. ‘Peers’ refers to NSP service users who the materials are aimed at. This commentary has been co-written with three of the five peers.

## Main body

### Methods

#### User-centred design process

**Fig. 1:**
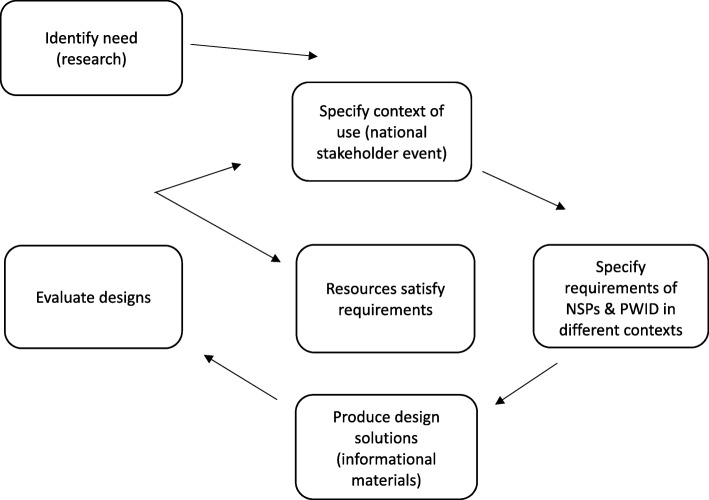
User-centred design process. Adapted from https://www.usability.gov/what-and-why/user-centered-design.html. Diagram displaying the user-centred design process followed in the project

We followed a flexible and iterative user-centred design process (Fig. 1) [25] in which we (1) identified the need for the information materials through our research (described above), (2) specified the context of use (identifying the audience for the materials), (3) specified the requirements of NSP and PWID in different contexts, (4) created design solutions with co-designers (or peers) to meet the needs of the target audience, and (5) evaluated and refined the designs with co-designers until they satisfied their needs.

##### Specifying context

We held a national meeting to discuss how we can accelerate the pace and scale of the rollout and uptake of detachable LDSS via NSP [26]. The meeting aimed to identify the key messages to be translated into communication materials and develop strategies to support fast implementation.

Before the event, we ran two discussion groups with Bristol Drugs Project (BDP) service users who had used detachable LDSS, and staff and volunteers working in NSP. We wanted to find out their experiences of the new equipment, recommendations for implementation, and suggestions for the content of communication materials to encourage the use of detachable LDSS. One of the service users, a BDP assertive engagement worker and National Institute for Health Research (NIHR) CLAHRC West’s Patient and Public Involvement Facilitator presented the feedback from the service user group at the stakeholder event. This process is in line with ‘planning and executing interventions’ (p20) capable of overcoming barriers to implementation described within the ‘Knowledge to Action Process’ [17].

We seconded an assertive engagement worker from BDP to the project; their specialist expertise and connections were vital to this project’s success. We felt it was important to involve PWID as co-designers of these harm reduction materials, to ensure they are relevant and appropriate.

##### Specifying requirements

Next, we ran a consultation process with eight NSP across the UK to understand barriers to the uptake of LDSS, and how different programmes operate and share harm reduction messages. This was to ensure that the materials would be useable in different NSP contexts and that the messages would be relevant. Graham and colleagues refer to this process as adapting the knowledge or research to the local context to ensure its appropriateness [17].

Most NSP visits were facilitated by our collaborators from Exchange Supplies who emailed invitations to NSP in a range of geographical areas with varying experiences of detachable LDSS. DB conducted all consultations and JK attended two. Consultations were mostly conducted face-to-face with one or a group of staff operating the NSP (apart from one which had to be carried out by phone due to poor weather conditions). A semi-structured topic guide was followed covering an overview of the NSP (e.g. opening hours, staff number, how the NSP operates), service user characteristics, key issues for services in this geographical area with this client group, harm reduction information provision to service users (e.g. what and how information is provided, what works well/less well), if and how detachable LDSS had been introduced and received, and feedback on the first draft of content for the materials. This informed a second draft of the content for the materials, which was itself developed from the original research and stakeholder event.

##### Produce design solutions and evaluate designs

We produced a design brief and invited proposals from designers. We selected Michael Linnell of Linnell Publications [27], an experienced designer specialising in harm reduction.

Five peers (three men and two women) known to BDP staff were invited to take part in the project. We looked for people who were likely to be willing and confident to express their views in a group setting. We tried to ensure the group was diverse in terms of gender, injecting experiences, attitude to detachable LDSS, and awareness of a wide range of harm reduction information and materials. However, the group was not ethnically diverse. One of the peers had attended the national stakeholder meeting (see above). Their involvement throughout the project was valuable as they had insight into its development and were able to share this experience with the other peers. All peers were paid with high street shopping vouchers for their input, following INVOLVE guidance [28].

**Fig. 2:**
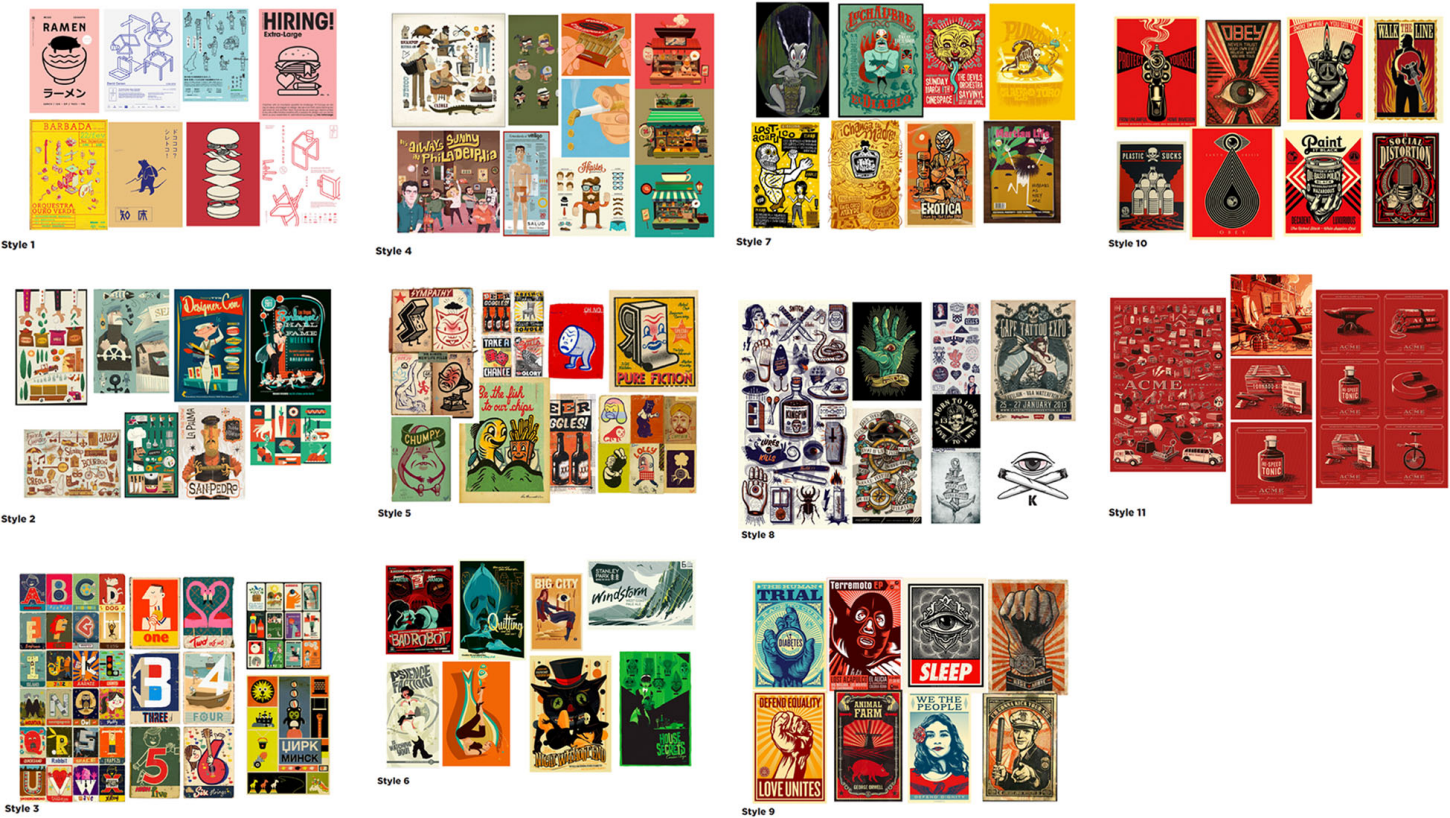
Style sheets. Eleven design style sheets created by the designer and presented to the peers in the first workshop. The peers selected style sheets 9 and 10

**Fig. 3:**
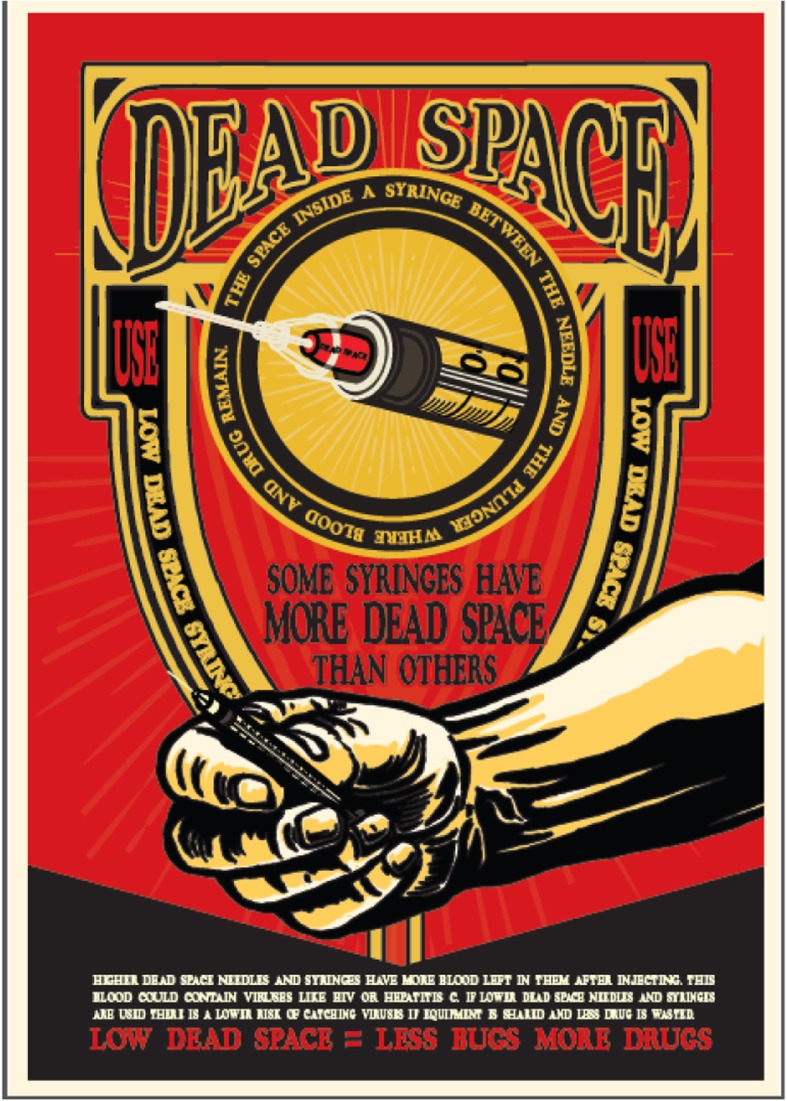
Initial design. One example of the first draft designs discussed in the second workshop

**Fig. 4:**
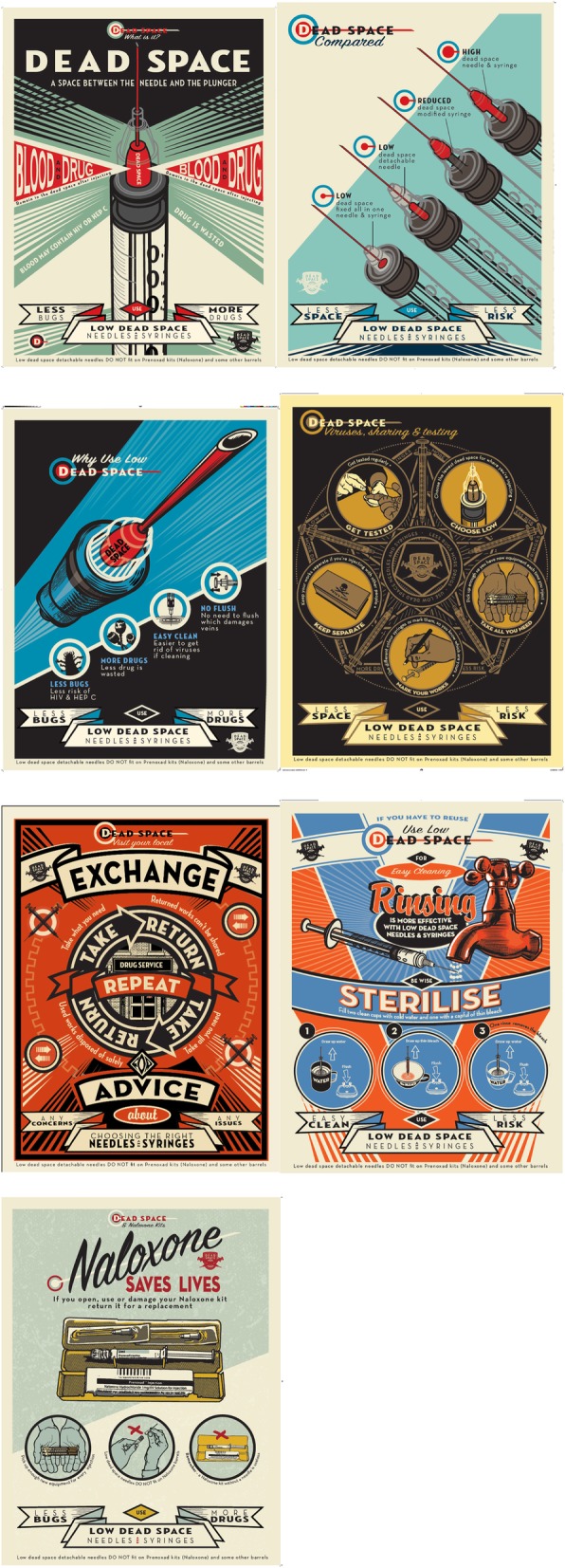
Final designs. The seven final designs available to download here are (https://www.exchangesupplies.org/shopsect_linnell_publications.php): ‘Dead space—what is it?’: this poster is designed to inform people about dead space, clearly illustrating what it is. ‘Dead space compared’: this poster shows the dead space in various types of injecting equipment, to help service users make an informed choice. ‘Less bugs, more drugs’: this poster displays the benefits of using low dead space equipment. These benefits were identified through the research. ‘Dead space, viruses, sharing’: this poster combines the message about choosing the lowest dead space possible for where people inject, with broader harm reduction messages. ‘Take, return, repeat’: this poster is designed to encourage the return of used equipment to needle and syringe programmes. ‘Be wise, sterilise’: this poster explains that rinsing low dead space equipment is more effective and gives the steps for sterilising equipment. ‘Naloxone saves lives’: this poster aims to encourage naloxone use, while increasing awareness that low dead space needles are not compatible with Prenoxad kits

We held three workshops to design the materials. In the first workshop, the peers selected their preferred design style from a range of options (Fig. 2). We then discussed their preferences for the tone of the materials, use of language and imagery, and use of corporate branding and content. In the second workshop, they were shown the first draft designs (Fig. 3) and asked to comment on the language, messages, and overall look and feel. The final workshop was an opportunity for peers to feedback on all the designs before they were finalised (Fig. 4).

A fourth reflective discussion workshop was held to reflect on peers’ and project facilitators’ (researcher, assertive engagement worker, and communications manager) experience of the project. In this group we posed the following questions to each other:

- What did peers see as their role in the project?
- What did peers get out of and learn from involvement in the project?
- What did the facilitators do that the peers liked/disliked?
- What have the facilitators learnt from involving peers in the project?

Detailed notes were taken by JK and ZTW at each workshop, incorporating all feedback, including contradictory points, and fed back to the designer—who was present at the final two of the three design sessions. We present a summary of the key reflections from each phase below.

### Reflections

#### National stakeholder meeting

Twenty stakeholders, including staff and service users from NSP, injecting equipment manufacturers, local authorities, commissioners, Public Health England, PWID, and academics, attended the meeting. These stakeholders discussed the intended audience and key messages and what could be achieved by producing a video and other communication materials. The stakeholders suggested that information materials with some animated content would be more appropriate than a video to reach service users who are the primary audience for these materials. Staff and volunteers working in NSP and commissioners are secondary audiences.

The group agreed that the content should go further than simply encouraging the use of detachable LDSS, by promoting broader injecting harm reduction messages.

The stakeholders agreed the following topics to cover:

1. Needle and syringe programme benefit
2. Equipment choice
3. Safer injecting practices
4. Encouraging the return of used equipment
5. Low dead space needles
6. Rinsing and sterilising equipment

#### NSP consultation

The NSP visited varied in terms of if and when detachable LDSS had been introduced, number of service users, number of staff, and size of city or town. The common issues they faced included the following:

- Service users being in a rush, so giving harm reduction advice was challenging;
- High use of pharmacy NSP, where less harm reduction advice was provided, rather than using specialist services;
- Staff who do not regularly work in NSP found providing harm reduction advice difficult;
- Low levels of equipment returns.

The response to introducing detachable LDSS varied. Most services had completely replaced HDSS equipment rather than phasing LDSS in gradually as recommended by our research [13]. While some reported no issues, others described service users requesting the old equipment and initial complaints about the change.

Overall, feedback on the proposed use and content of the information materials was positive. Key feedback included ensuring images of equipment were generic rather than brand specific, and including a clear explanation of which equipment is appropriate for service user needs and why. NSP also wanted the materials to include messages encouraging the return of used equipment and rinsing used equipment and messages emphasising the benefits of using specialist NSP (rather than community pharmacy) including confidentiality, continuity of staff, and the ability to build trusting relationships.

This consultation provided key insights for the next phase of developing the materials.

#### Co-design workshop 1: Choosing the design styles and considering appropriate language

##### Retro and unique style selected

Discussing the style sheets was revealing both in terms of the peers’ preferences, but also what they did not like. For example, they considered a cartoonish style patronising and not serious enough.

The peers selected a strong retro Soviet propaganda style (Fig. 2 styles 9 and 10) using a simple contrasting colour palette of red, black, and cream. They chose this style because it was unlike the style of traditional harm reduction materials, targeted at PWID bold, bright, and eye-catching and drew the eye to the centre of the design. Peers thought these design features would help attract attention and encourage people to adopt the practices communicated in the messages.

##### Avoiding stereotypical imagery

Peers emphasised the importance of avoiding images with negative connotations, which could be interpreted as implying PWID are ‘disgusting’ or ‘dirty’, such as very veiny arms or shocking images of health problems. Line or diagrammatic drawings struck the right tone of being serious while not carrying any value judgement. The peers preferred images of body parts, such as arms or hands, rather than whole people. This also meant images could be gender neutral. Similarly, the group did not like images of eyes which give a sense of being watched, looked down on, and judged or of pointing fingers which felt accusatory.

The peers recommended avoiding stereotypical portrayals of PWID (e.g. homeless people) because they could cause offence and lead to people feeling stigmatised or judged. The peers discussed that people may feel disconnected from images to which they cannot relate. Images representing a small portion of PWID may result in people feeling the information is not intended or useful for them.

##### Ensuring appropriate language and tone

Peers felt it was important to avoid unequivocal messages which tell people what to do, especially messages about the dangers of using drugs. This could be patronising, would not be appropriate to the target audience, and could lead to people rejecting all the messages. Universal slang terms (e.g. ‘works’) were preferred over more generic, factual language (e.g. equipment), but local slang should be avoided.

The group suggested the use of humour should be carefully considered—everyone felt that a serious tone should be adopted and that humour had the potential to get in the way of clear messages and trivialise a serious topic. The peers knew people who had HCV and HIV, and they felt that messages should reflect the seriousness of the issues.

##### Content

The message that the new equipment would save the NHS (United Kingdom National Health Service funded by the government to provide medical and health care services) money due to reduced HCV treatment costs [29] did not resonate with most of the peers. Most also viewed the use of NHS and NIHR (which funds health and social care research in England) logos on materials negatively as this could imply blaming PWID for healthcare costs. Some peers also expressed a mistrust of the NHS, having received poor treatment and feeling that health professionals did not care about them or understand their experience.

#### Co-design workshop 2 and 3: Feedback on the designs

In response to the initial designs (Fig. 3), there was a general preference for images without body parts. During the first workshop, the peers agreed that body parts should be gender neutral. However, when they saw the initial designs, the group felt the arm image (Fig. 3) would be perceived to be male. One peer pointed out that there is no such thing as a ‘gender neutral’ body part. Even images intended to be neutral are often interpreted as male*—*‘for example, female toilet signs have skirts to indicate the gender!’, At this point, it was agreed that it was too difficult to design body parts that reflect everyone, and as the messages were about the equipment, service users would make the connection between dead space and blood without body images. Although they acknowledged that the initial designs may reflect some ‘truth’ or accuracy, the peers explained that people do not want to be confronted with this and the peers did not want to contribute to the stigmatisation of others.

From this process, seven attractive and engaging posters (Fig. 4) were produced. The first poster is designed to inform people about dead space. The peers selected the strapline ‘Less bugs, more drugs’ which was also one of the key messages from the original research. The second poster displays the dead space in the various types of injecting equipment to help service users make an informed choice about their equipment. This was the most challenging poster to get right visually and in terms of the language used. The next poster highlights the benefits of using low dead space equipment. We designed the poster about rinsing and sterilising to emphasise that low dead space equipment is more effective to clean because there is less space between the needle and plunger. This poster also provides the steps for sterilising equipment and highlights the importance of cleaning LDSS before re-using. The ‘Dead space, viruses, sharing’ poster combines the message about choosing the lowest dead space possible for the injection site with broader harm reduction messages, including getting tested for BBVs, and taking enough equipment from NSP. The ‘Take, return, repeat’ poster encourages the return of used equipment to NSP. Low equipment return rates was an issue highlighted during the visits to NSP. The last poster was not part of our original plans. It was designed to increase awareness that new low dead space needles are incompatible with the barrels in Prenoxad kits containing naloxone. If the needles from the naloxone kit are used to inject drugs, the kit must be returned for a replacement. Otherwise, the kit will be useless when people need to administer naloxone. This was highlighted as an issue by the BDP members of our steering group.

These poster designs were also reworked into a 16-page booklet (https://www.exchangesupplies.org/shopsect_linnell_publications.php) and a series of short animations (https://binged.it/2INd7TY), which were identified as useful formats during the NSP consultation stage.

#### Co-design workshop 4: Peer and facilitator reflections on the project

##### Co-designer role: consultant and level of anticipated vs actual involvement

One of the group commented that the explanation of the project’s purpose and their role was clear and they felt supported to attend and contribute. Peers viewed themselves as consultants to the project and liked the idea of being involved. One co-designer initially thought their role would be more tokenistic, expecting to be shown the materials once they had been designed and asked to provide feedback on them (e.g. ‘Do you understand these materials? Do you like them?’). Rather than being ‘done’ to them, the peers talked about feeling fully involved in the entire process.

One peer commented that most information materials available in NSP are not really looked at because they are too plain. They suspected they probably had not been made by service users and did not appear to ‘have them in mind’. By involving peers in this project, they felt the materials were more credible as they reflected the language used by service users and their preferences.

##### Positive experiences

The peers enjoyed being involved in the design process, from choosing style sheets which best represented them to seeing the designs evolve over time. Importantly, the peers felt their feedback was incorporated at every stage of the design process. For example, the group decided on the order of the benefits of LDSS and the language used, preferring ‘less bugs, more drugs’ to ‘more bang for your buck’. They felt the latter was American in tone, overly familiar and likened it to when Google says ‘Oops!’ when it crashes—‘I’m not your friend Google!’ Other examples were not using body part imagery and avoiding humour.

Participants said that having their voices heard and valued was a validating experience, contrasting with some previous experiences of participating in research (‘You can feel like a rat in a cage sometimes, with people coming in to study you’).

### What was learnt from involvement in the project?

#### Awareness of and attitude towards LDSS

Throughout the project, the group became more aware of the concept of dead space and the benefits of using the lowest dead space equipment possible. One person had not liked detachable LDSS at the start of the project and found them to be flimsier than the high dead space equivalent. However, having spoken to the equipment developer at the stakeholder event, they were convinced of their benefits and made the switch. Another person was not aware of why low dead space was important but now understands it well and will continue to use LDSS. Lastly, two of the group did not like LDSS because the coloured needle hub makes it difficult to know if you are in a vein. Participating in the group did not change their view on this, but one of these peers felt they had learned enough about the equipment to know that they would be useful if their lifestyle was more chaotic and they were more at risk of sharing equipment.

#### Awareness of broader harm reduction issues

Some other beneficial learning from participation in the project included how to rinse and sterilise the equipment effectively, that low dead space needles do not fit on naloxone barrels, and that flushing damages veins. As a result, one member talked about sterilising needles as a new habit and two had tried to reduce the amount they flush.

#### Sharing learning with others

The peers were motivated to share harm reduction messages learnt through the project with others. They spoke to other people about the project, dead space, and broader harm reduction messages like cleaning equipment after use, not re-using, or flushing.

### Practicalities

Running the group in the afternoon and payment for their time supported engagement and was appreciated.

### Group composition and dynamics

The group felt they had a good mix of experiences, knowledge, and ages. They worked well together, were respectful of each other, and listened to differing opinions.

The peers felt that the designer and facilitators listened to them and accepted their views. There was no condescension or sense of being judged, and the designer and facilitators guided and helped crystallise their ideas. The designer had ‘no airs and graces’, his tone and body language were engaging, and he clearly wanted to hear what they had to say. He was not precious about his work, and there was an understanding that everyone shared the same goal.

### What have the facilitators learnt from involving peers in the project?

#### Benefits of peer involvement

All facilitators agreed that it has been invaluable and rewarding to collaborate with peers in this work. Without working with peers, the materials would have been very different and likely ineffective at communicating and engaging with the audience. For example, they may have contained depictions of cartoon characters, body imagery, and humour all of which had the potential to stigmatise and result in a negative response. The discussion around body imagery and gender neutrality also highlights the importance of prolonged engagement with peers because it was only by seeing the designs that the group could determine it was not appropriate. More broadly, this project has led to greater recognition across BDP of the benefit of service user involvement in service design and delivery.

#### Forming equitable partnerships

The facilitators sought to create an equitable partnership with the peers in several ways. Firstly, they placed greater emphasis on the peers’ preferences than their own. At the first workshop, the facilitators emphasised that the peers were the experts and that their input would be taken seriously.

During the workshops, the facilitators made a conscious effort not to interject, use closed or leading questions, or give personal views while maintaining a collaborative, encouraging approach. However, it was difficult at times not to intervene or unintentionally influence the views of the co-designers as the facilitators had spent a considerable amount of time considering the content for the materials and were also very passionate about the project.

When getting feedback from the project steering group, the facilitators advocated for the peers. They consciously kept the peers’ views in mind, emphasising and reiterating them when necessary. In this way, the peers were given a voice even in conversations where they were not present.

Table 1 presents key learning outcomes from this project.

**Table 1:** Key learning outcomes. This table presents learning outcomes and recommendations from the project

Learning outcome topic	Description	Recommendation
Stakeholder consultation is key	The consultation process with NSP ensured the informational materials are useable/relevant in different NSP contexts.	Identify and be responsive to the needs and requirements of a broad range of stakeholders.
Importance of emphasising peers’ preferences	The facilitators emphasised that the peers were the experts in the co-design process.	Clarify roles and form equitable partnerships at the beginning of the project.
Need to recognise threats to co-production	The facilitators found it difficult at times not to intervene or unintentionally influence the views of the co-designers.	Engage in reflective practices to recognise and overcome threats to equitable co-production.
Necessary to give peers’ a voice	The facilitators advocated for peer viewpoints during meetings they did not attend.	Voice peer viewpoints when they are not present.
Flexibility and reflection	This project highlights the importance of being flexible and responsive to new insights and ideas.	Co-designed projects require flexibility in their approach to ensure the product produced meets the end users’ needs.

### Strengths and Limitations

This project has a number of strengths and limitations that warrant consideration. We did not follow a formal process for capturing each stage of this project. For example, we did not formally analyse the reflections using a theoretical framework. Including peers who were expected to actively engage in the workshops may mean that the materials were not reflective of all service users. The group did reflect on the anticipated views and experiences of others, including those with less settled lifestyles. We feel that, on balance, the group were well placed to contribute to this project. The group was not ethnically diverse, though this reflects the BDP service user population. The consultation process helped ensure the content of the materials is relevant to different NSP contexts and our approach is in line with guidance on co-producing research [24].

## Conclusions and recommendations for future co-design projects

As there is no single approach for performing co-produced projects [24], this commentary serves as an example of how research findings can be implemented through the co-design of information materials. Placing peers at the heart of this project was essential to ensuring the materials were appropriate and engaging and avoid unintentionally stigmatising or alienating the intended audience. We recommend that others planning similar work include peers in the entire project to support their meaningful contribution.

The facilitators approached the project with an open mind and advocated for the views of the peers, whose opinions were paramount. The professionals involved in such projects must practice humility and be prepared to have their views challenged, such as the use of humour in drug harm reduction materials, which is a well-established tradition.

Harm reduction materials that resonate with their intended audience are more likely to influence those people’s attitudes and behaviour. The peers’ contribution was central to developing materials that were appealing while treating the intended audience with respect.

## Abbreviations

BBV: Blood-borne virus
BDP: Bristol Drugs Project
HCV: Hepatitis C virus
HDSS: High dead space syringes
HIV: Human immunodeficiency virus
LDSS: Low dead space syringes
NSP: Needle and syringe programmes
PWID: People who inject drugs
